# High prevalence of human immunodeficiency virus, hepatitis B and C viral infections among people who inject drugs: a potential stumbling block in the control of HIV and viral hepatitis in Tanzania

**DOI:** 10.1186/s12889-020-8294-8

**Published:** 2020-02-04

**Authors:** Rahim H. Kawambwa, Mtebe V. Majigo, Ahmed A. Mohamed, Mecky I. Matee

**Affiliations:** 1 Department of Microbiology and Immunology, Muhimbili University of Health and Allied Sciences, Dar es Salaam, Tanzania; 2 Field Epidemiology and Laboratory Training Programme, Dar es Salaam, Tanzania

**Keywords:** PWID, HIV, Hepatitis B virus, Hepatitis C virus, Dar es Salaam, Tanzania

## Abstract

**Background:**

Tanzania has witnessed a significant decrease in the prevalence of human immunodeficiency virus (HIV) and viral hepatitis in the general population attributed to several interventional measures. It is uncertain whether this decline has also occurred among people who inject drugs (PWID). This study aimed to determine the seroprevalence of HIV, Hepatitis B and C viruses infection among PWID recruited from their hotspot sites in Dar es Salaam, Tanzania.

**Methods:**

A cross-sectional study conducted between June and September 2017 recruited PWID from pre-identified hotspot sites using a snowball referral sampling technique. A structured questionnaire was used to obtain information regarding socio-demographic characteristics, behaviour and drug use. Blood was tested for the presence of IgG antibodies against HIV and Hepatitis C virus (HCV) and hepatitis B surface antigen (HBsAg). Data were entered in the computer using excel software and analysed using Statistical Package for Social Sciences version 20.

**Results:**

A total of 219 PWID were recruited, the majority of whom were males (74.9%), unmarried (60.7%), had low education (62.6%) and low income (57.1%). The median age was 39 years, with an inter-quartile range of 35–43. Approximately 32.0% had a history of drug injection for more than 3 years, 79.9% were injecting drugs more than 3 times per day and 47.5% were sharing needles. The overall prevalence of HIV, HBsAg, and HCV was 33.8, 7.8, and 50.2%, respectively. There was serologic evidence of at least one infection for 68.9%, while 22.4% had two or more infections. HIV infection was independently associated with being married, while HCV was associated with injecting drugs for more than 3 years and unprotected sex.

**Conclusion:**

Over two-third of PWID had serologic evidence of infection with at least one virus while 22.4% having at least two infections. The high prevalence of HIV and viral hepatitis infections among PWID may hamper initiatives of ending HIV and viral hepatitis epidemics in Tanzania.

## Background

Tanzania has reported a steady fall in the prevalence of human immunodeficiency virus (HIV) infection in the general population, from 7% in 2004 [1] to 5.0% in 2017 [2]. The prevalence of hepatitis B and C viruses in the general and some sub-populations like health care workers, pregnant women and children have also been reported to decline [3, 4]. These decline have been attributed to several preventive measures such as increasing awareness; screening blood donors; free condom distribution; prevention of mother to child transmission; expanded access to antiretroviral therapy; voluntary male circumcision; setting up HIV care and treatment clinic and methadone clinics [2]. It is unclear whether these preventive measures have had an effect on people who inject drugs (PWID) [5–8].

Injecting drug use (IDU) has been associated with increased risk of HIV, HBV, and HCV infections [9–12]. The prevalence of IDU vary greatly between countries, ranging from 0·02% to 5·21% [9]. A few studies conducted in Africa have reported IDU to be on the rise with potential significant contribution to the occurrence and spread of blood-borne infections [12–14].

In Tanzania, IDU has been reported since the 1990s [14]. Recognizing the scale of PWID, primarily the use of heroin [14] and high prevalence of HIV infection among them [1], a special outreach program was established in 2007, that offers a variety of preventive and supportive services including; education and communication materials, psychosocial support services and family group therapy meetings [15]. However, it is unclear as to whether these interventions, among several others taken by different stakeholders, have had an effect on the prevalence of the major blood-borne viral infections of public health importance. We conducted this study to provide the current status of seroprevalence of HIV, HBV and HCV infections among PWID in Dar es Salaam, recruited from their hotspot sites.

## Methods

### Study design and setting

This was a cross-sectional study conducted between June and September 2017 in Dar-es-salaam, the largest city in Tanzania with an estimated population of around 6 million inhabitants. Participants were recruited from pre-identified hotspot sites locally known as ‘maskani’ in Sinza, Kinondoni, Kimara, Tandale, Msasani, Mbagala, Kunduchi, Temeke, and Tandika suburbs of the City.

### Study population

PWID aged 18 years or older, not enrolled in a methadone program, were eligible for inclusion into the study, while recruitment was subject to obtaining written consent. An individual had to admit having injected non-medically sanctioned psychotropic substances such as opioids, amphetamine-type stimulants, cocaine, hypo-sedatives, and hallucinogens either intravenously, intramuscularly or subcutaneously, during the past 30 days. The sample size was estimated to be 155 using Kish Leslie formula, and considering an 11∙3% prevalence of HIV among PWID in Zanzibar [6] and a 5% margin of error.

### Recruitment and data collection

Participants were recruited following snowball chain referral sampling. First, we identified 30 PWID attending Muhimbili National Hospital (MNH) Methadone Clinic, who were requested to link researchers to PWID at hotspot sites. The selection of initial participants and their respective sites was based on information provided by 30 contacts at MNH methadone. At the hotspot site, the research assistant first introduced the study and those consented to join were recruited after signing a written consent. After completion of the interview and specimen collection, each participant was asked to refer other PWID to the study, continuously until the predetermined sample size was reached. A structured questionnaire was used to collect information related to social demographic characteristics, risk behavior, drug use habits and status of HIV, HBV and HCV (Additional file 1).

### Specimen collection

Trained research assistants using plain vacutainer tubes drew about 4 ml of venous blood from each participant aseptically, which were transported every day in cool boxes to the Muhimbili University of Health and Allied Sciences (MUHAS), Microbiology Laboratory for processing.

### Laboratory assays

Blood was tested for HIV infection using SD Bioline HIV-1/2 3.0 (Standard Diagnostic, Seoul, Korea), and reactive samples were confirmed using Uni-GOLD HIV (Trinity Biotech, Wicklow, Ireland). HBsAg rapid test strip (Standard Diagnostic, Seoul, Korea) was used for the detection of hepatitis B surface antigen (HBsAg). The presence of HCV infection was determined by the detection of IgG antibodies to HCV using an anti-HCV rapid test strip (Standard Diagnostic, Seoul, Korea).

### Data analysis

Data were entered in excel software and analysed using Statistical Package for Social Sciences (SPSS) version 20.0. Categorical variables were summarized as proportions while continuous variables were summarized as median with inter-quartile range. The seroprevalence of HIV, HCV, and HBsAg among PWID was expressed in percentages and comparisons between the groups were done using Chi-Square. Low income was defined as earning less or equal to 45 US dollars per month. Binary simple logistic regression was first performed, followed by a multivariable logistic regression model to determine factors independently associated with HIV, HBV or HCV infection. All variables with *p*-value < 0.2 in simple binary regression were included in the multivariable model. Associations in the multivariable logistic model were presented as adjusted odds ratios (AOR) with 95% confidence interval (CI). Interactions between independent variables were examined, and the Wald test was used to test the associations of the variables and interactions. The Hosmer-Lemeshow test was used to examine the overall fitness of the model. The level of significance was specified at 0.05.

## Results

### Participants’ characteristics

The study recruited a total of 219 PWID. Around 2% of PWID were eligible but did not provide written consent. The median age of the participants was 39 years; [Inter-quartile range 35–43 year] (Table 1). The majority were males (74.9%), unmarried (60.7%), had basic primary education (62.6%) and 57.1% earned less or equal to 45 US dollars per month. Approximately 32.0% had a history of drug injection for more than 3 years, 79.9% were injecting drugs more than 3 times per day, while 47.5% were sharing needles. More than half (51.1%) either never used or used condom irregularly in the last 6 months (Table 1). None of the participants knew status of infection with any of the three viruses before enrolment in the study.

**Table 1:** Socio-demographic and behavioral characteristics of 219 PWID in Dar-es-salaam

Variables	Frequency (n)	Percentages (%)
Age group (yrs)		
< 39	105	47.9
≥ 49	114	52. 1
Sex		
Male	164	74.9
Female	55	25.1
Marital status		
Unmarried	133	60.7
Married	86	39.3
Education level		
Low education	137	62.6
High education	82	37.4
Occupation		
Unemployed	102	46.6
Employed	54	24.7
Business	63	28.8
Monthly income (USD)		
≤ $45	125	57.1
> $45	94	42.9
Duration of injection		
≤ 3 years	149	68.0
> 3 years	70	32.0
Frequency of injection per day (last 6 months)		
≤ 3 times	44	20.1
> 3 times	175	79.9
Needle sharing (last 6 months)		
No	115	52.5
Yes	104	47.5
Use of condom (last 6 months)		
Never/Irregular	112	51.1
Always	107	48.9

### Prevalence of HIV, HBV, HCV, and co-infection

The overall seroprevalence of HIV, HBV and HCV was 33.8% [27.8–40.5], 7.8% [4.9–12.1] and 50.2% [43.7–56.8], respectively. One hundred and fifty-one (68.9%) of PWID had serologic evidence of at least one infection, 49 (22.4%) had at least two infections, while one (0.5%) had serologic evidence of all the three viruses (Table 2). Considering co-infection, 0.9% had HIV + HBV, 16% had HIV + HCV and 6.4% had HBV + HCV. Among participants with HBV infection, 82.4% had also HCV (Table 2). There was no sex-stratified significant difference observed regarding the occurrence of HIV, HBV and HCV infections.

**Table 2:** Seroprevalence of HIV, HBV, HCV, and co-infections among 219 PWID in Dar-es-salaam, Tanzania

Infection	Frequency positive	Seropositive	95% CI of seropositive
HIV Antibody	74	33.8	27.8–40.5
HBV surface antigen	17	7.8	4.9–12.1
HCV antibody	110	50.2	43.7–56.8
HIV + HBV	2	0.9	0.3–3.3
HIV + HCV	35	16.0	11.7–21.4
HBV + HCV	14	6.4	3.8–10.4
HIV + HCV + HBV	1	0.5	0.1–2.5
Any infection	151	68.9	62.5–74.7

### Factors associated with HIV, HCV and HBV infections

Factors associated with the occurrence of HIV, HCV, and HBV are summarized in Table 3. HIV infection was significantly higher among those who were married, sharing needles and inconsistent users of condoms (*p* < 0.05), while HCV was associated with drug injection for more than 3 years, and lack or irregular use of condoms (*p* < 0.05). HBV was not associated with any of the risk factors evaluated (*p* > 0.05) (Table 3).

**Table 3:** Proportion of HIV, HCV, and HBV seropositive in relation to participants’ characteristics

Variables	HIV		HCV		HBV	
	Pos (%)	*p*-value	Pos (%)	*p*-value	Pos(%)	*p*-value
Age group (yrs)		0.89		0.73		0.15
< 39	35(33.3)		54(51.4)		11 (10.5)	
≥ 39	39(34.2)		56(49.1)		6 (5.3)	
Sex		0.13		0.26		0.38
Male	60(36.6)		86(52.4)		11(6.7)	
Female	14(25.5)		24(43.6)		6(10.9)	
Marital status		0.01		0.25		0.39
Unmarried	36(27.1)		63(47.4)		12(9.0)	
Married	38(44.2)		47(54.7)		5 (5.8)	
Education level		0.33		0.74		0.08
Low education	43(31.4)		70(51.1)		14 (10.2)	
High education	31(37.8)		40(48.8)		3 (3.7)	
Occupation		0.39		0.58		0.98
Unemployed	37(36.3)		55(53.9)		11(10.8)	
Employed	20(37.0)		26(48.1)		6(11.1)	
Business	17(27.0)		29(46.0)		0 (0.0)	
Monthly income (USD)		0.17		0.63		0.51
≤ $45	47(37.6)		61(48.8)		11 (8.8)	
> $45	27(28.7)		49(52.1)		6 (6.4)	
Duration of injection		0.18		0.01		0.053
≤ 3 years	46(30.9)		66(44.3)		8 (5.4)	
> 3 years	28(40.0)		44(62.9)		9 (12.9)	
Frequency of injection per day (last 6 months)		0.76		0.48		0.12
≤ 3 times	14(31.8)		20(55.5)		6(13.6)	
> 3 times	60(34.3)		90(51.4)		11 (6.3)	
Needle sharing (last 6 months)		0.03		0.84		0.59
No	31 (27.0)		57(49.6)		10(8.7)	
Yes	43(41.3)		53(51.0)		7 (6.7)	
Condom use (last 6 months)		0.02		0.004		0.39
Never/irregular	46(41.1)		67(59.8)		7 (6.2)	
Always	28(26.2)		43(40.2)		10 (9.3)	

Key: *Pos* Positive

Multivariable logistic regression model was performed to measure the adjusted relationship between independent variables and occurrence of HIV, HCV, and HBV. Marital status was the only factor independently associated with HIV infection (AOR 2.04, 95%CI -1.10 – 3.79), while HCV infection was associated with drug usage for more than 3 years (AOR; 2.04, 95% CI – 1.11- 3.74). PWID who used condom regularly decreased odds of getting HCV infection (AOR = 0.42, 95% CI – 0.22 – 0.79) (Table 4).

**Table 4:** Association of participants’ characteristics with the adjusted odds of seropositive for HIV, HBV, and HCV among PWID in Dar-es-salaam

Variable	HIV		HCV		HBV	
	COR (95% CI)	AOR (95% CI)	COR (95% CI)	AOR (95% CI)	COR (95% CI)	AOR (95% CI)
Age > 39 yrs	1.04(0.59–1.82)	^a^	0.91(0.54–1.55)	^a^	0.48(0.17–1.33)	0.56(0.17–1.79)
Male Sex (Ref. Female)	0.59(0.29–1.74)	0.62(0.29–1.30)	0.70(0.38–1.29)	0.93(0.48–1.88)	1.70(0.60–4.84)	1.51(0.42–5.39)
Married Marital status (Ref. Unmarried)	2.13(1.20–3.78)	2.04(1.10–3.79)	1.34(0.78–2.31)	1.03(0.57–1.86)	0.62(0.21–1.83)	0.66(0.19–2.26)
High education level (Ref. Low level)	1.33(0.75–2.36)	^a^	0.91(0.53–1.58)	^a^	0.33(0.09–1.20)	0.31(0.08–1.25)
Employed (Ref: Unemployed)	1.03(0.52–2.05	^a^	0.79(0.41–1.54)	^a^	1.04(0.36–2.97)	^a^
Business (Ref: Unemployed)	0.65(0.33–1.29)	^a^	0.73(0.39–1.37)	^a^	^b^	^a^
Monthly Income>45USD	0.67(0.37–1.19)	0.58(0.32–1.06)	1.14(0.67–1.95)	^a^	0.71(0.25–1.98)	^a^
IDU > 3 years	1.49(0.83–2.69)	1.31(0.69–2.46)	2.13(1.89–3.81)	2.04(1.11–3.74)	2.60(0.96–7.06	2.42(0.72–8.12)
Injection > 3 times per day	1.12(0.55–2.27)	^a^	1.27(0.65–2.47)	^a^	0.43(0.15–1.22)	0.72(0.19–2.72)
Needle sharing	1.91(1.08–3.37)	1.75(0.92–3.29)	1.06(0.62–1.79)	0.76(0.41–1.39)	0.76(0.27–2.07)	0.70(0.21–2.35)
Use of condom	0.51(0.29–0.90)	0.76(0.39–1.48)	0.45(0.26–0.77)	0.42(0.22–0.79)	1.55(0.57–4.22)	1.26(0.34–4.71)

Key: *AOR* Adjusted odds ratio, *CI* Confidence interval, *COR* Crude odds ratio, *Ref* Reference

^a^Not included in multivariable models

^b^Exncluded from multivariable medole because of zero observation in one cell

Potential interactions between marital status and condom use on HIV status was checked. The assumption was that the effect of condom use on HIV status is somewhat dependent on marital status. The *p*-value for interaction term was 0.67 which is far away from significance level and there was no much difference between the model with interaction term and the preliminary main effects model. Based on these observations we drooped the interaction term in the final model. The Hosmer-Lemeshow test result was *p =* 0.147, which indicated the fitness of the overall model.

## Discussion

Our study shows that over two-thirds of PWID in this study had serologic evidence of infection with at least one virus, while 22.4% having dual or triple multiple infections. About one-third of the participants were HIV positive, half had evidence of HCV infection and 8% HBV infection. These findings are higher in comparison with latest systematic review of studies conducted in sub-Saharan Africa, showing estimates of 21.8% for HCV, 18.3% for HIV and 3.7% for HBV [12]. Our findings are also higher compared with the previous report from Tanzania [6], possibly indicating an increase of infection with these three viruses among PWID. The rate of infections with these three viruses has shown a steady rise [5, 7, 16]. The findings indicate that PWID is a key population that needs urgent attention as a strategy to achieve control of HIV and viral hepatitis infections.

Alarmingly none of our study participants knew their status of infection with any of the three viruses before enrolment, which may indicate limited access to testing services. The higher prevalence of the three viruses among PWID compared to previous studies could be attributed, at least in part, due to a number of factors such low access to testing services and therefore not being able to access care and treatment services, multiple sexual partners and high frequency of partner change with low condom usage [6]. Indeed, regular condom use was associated with a 60% decreased odds of having HCV infection among those reported regular use of condom.

The association between condom use and HCV infection is interesting with the fact that HCV is not typically sexually transmitted. Previous studies have provided evidence that sexual transmission of HCV within low-risk heterosexual partnerships occurs at most with very low frequency [17]. However, in a population where there is a large pool of HCV infected persons like what our study found and sexual intercourse is common, even a low rate of sexual transmission can account for a large number of new cases. This may explain the decreased odds of HCV infection among those reported regular use of condom.

In the univariate analysis model, consistent condoms use showed a negative association with HIV infection, while marriage showed a positive association. However, in multivariable analysis condom use was not independently associated with HIV status. There was no evidence from the analysis to explain the absence of association of condom use and HIV infection in the adjusted analysis. The assumption that marriage could have an effect on the use of condom was not supported by the multivariable logistic regression model which included interaction term of marital status and condom use. Conversely, other previous studies have shown low condom usage among married couples [8, 16].

Our findings seem to indicate significant challenges in dealing with PWID that have certainly limited the impact of the initiatives undertaken by the Tanzania AIDS control program, including the outreach services. We recommend targeting PWID in their hideouts given illegality and stigma associated with drug use. The emphasis should be on social and psychological support, aiming at reducing high-risk behaviour, as well as increased access to HIV and viral hepatitis testing services and enrolment to antiretroviral and methadone schemes, which have shown clear benefits [18, 19]. We did not observe a significant relationship between monthly income and needle sharing practice, supporting findings of previous studies conducted in Tanzania [14, 16, 20], and disputing a common belief in the society that shared injection for drug use is due to financial constraints.

Our study may have several limitations. We acknowledge that reliance on self-reported information and dependency on the willingness of PWID to participate may have introduced selection and information biases and clustering of events and failure to recall. Reporting error may have occurred for some measures such as the frequency of injection and condom since this was based on participants’ ability to recall events in the last 6 months. HBV vaccination status was not evaluated and therefore the risk of acquiring HBV infection cannot be determined. Finally, the snowball sampling approach employed in this study may have resulted in a biased sample that cannot be corrected by weighting schemes, limiting inferences of our findings.

## Conclusion

The high prevalence of HIV, HBV, and HCV infections among PWID may hamper efforts to control these viruses in Tanzania. PWID should be actively screened at hotspots and linked to friendlier and more tailored care and treatment services for HIV, HBV, and HCV.

## Supplementary information

**Additional file 1.** Interview questionnaire.

## Data Availability

All relevant data generated and analysed during this study are available from the corresponding author on reasonable request.

## Abbreviations

CI: Confidence interval
HBsAg: Hepatitis B surface antigen
HBV: Hepatitis B Virus
HCV: Hepatitis C Virus
HIV: Human immunodeficiency virus
IDU: Injecting drug use
MNH: Muhimbili National Hospital
MUHAS: Muhimbili University of Health and Allied Sciences
PWID: People who inject drugs
