# Supplementary material for: High prevalence of human immunodeficiency virus, hepatitis B and C viral infections among people who inject drugs: a potential stumbling block in the control of HIV and viral hepatitis in Tanzania
Source: BMC Public Health. 2020 Feb 4;20:177. doi: 10.1186/s12889-020-8294-8 (PMC7001263; doi:10.1186/s12889-020-8294-8)
Supplement: Supplementary file 1 — Additional file 1. Interview questionnaire. [file 12889_2020_8294_MOESM1_ESM.doc]

**INTERVIEW QUESTIONNAIRE**

**HBV, HCV AND HIV INFECTION SEROPREVALENCE AMONG PEOPLE WHO INJECT DRUG IN DAR-ES-SALAAM, TANZANIA**

**Identification number [______________________]**

***“I’d like to thank you for taking part in this interview. All the information you give me will be confidential and your name won’t be recorded anywhere. The answers to some questions may seem obvious to you, but I need to ask you all of the questions. You are free to end this interview session at any point if you feel uncomfortable”***

| **General Information** | | | | | | | | | | | | | |  | |
| --- | --- | --- | --- | --- | --- | --- | --- | --- | --- | --- | --- | --- | --- | --- | --- |
| Location and Date | | | Response | | | | | | | | Code | | |  | |
| Name of District | | |  | | | | | | | |  | | |  | |
| Name of Location |  | | | | | | | |  | | |  | | | |
| Interviewer ID | └─┴─┴─┘ | | | | | | | |  | | |  | | | |
| Date of the interview | └─┴─┘ └─┴─┘ └─┴─┴─┴─┘  dd mm year | | | | | | | |  | | |  | | | |
| Consent, Interview Language and Name | | | Response | | | | | | | | Code | | |  | |
| Consent has been read and obtained | | | | Yes | 1, | | |  | | | | | | | |
|  | | | | No | 2 If NO, END | | |  | | | | | | | |
| Interview Language | | | | English | 1 | | |  | | | | | | | |
|  |  |  |  | Kiswahili | 2 | | |  | | | | | | | |
|  |  |  |  | Others | 3*[Insert Language]* | | |  | | | | | | | |
| Time of interview (24 hour clock) | | | └─┴─┘: └─┴─┘  hrs min | | | | | | | |  | | |  | |
| **Participant Demographic Information** | | | | | | | | | | | | | | |  |
| **Question** | | | **Response** | | | | | | | | **Code** | | | |  |
| 1.Sex (*Record Male / Female as observed)* | | | Male | | | | 1 | | | |  | | | |  |
|  |  |  | Female | | | | 2 | | | |  |  |  |  |  |
| 2. What is your date of birth? *Don't Know 77* | | | └─┴─┘ └─┴─┘ └─┴─┴─┴─┘  dd mm year | | | | | | | |  | | | |  |
| 3. How old are you? | | | Years | | | | └─┴─┘ | | | |  | | | |  |
| 4. In total, how many years have you spent at school and in full-time study (excluding pre-school)? | | | Years└─┴─┘ | | | |  | | | |  | | | |  |
| 5. What is the **highest** level of education you have completed? | | | No formal schooling | | | | 1 | | | |  | | | |  |
|  |  |  | Less than primary school | | | | 2 | | | |  |  |  |  |  |
|  |  |  | Primary school | | | | 3 | | | |  |  |  |  |  |
|  |  |  | Secondary school | | | | 4 | | | |  |  |  |  |  |
|  |  |  | High school | | | | 5 | | | |  |  |  |  |  |
|  |  |  | College/University | | | | 6 | | | |  |  |  |  |  |
|  |  |  | Post graduate degree | | | | 7 | | | |  |  |  |  |  |
|  |  |  | Refused | | | | 88 | | | |  |  |  |  |  |
|  | | | Don’t Know | | | | 77 | | | |  | | | |  |
| 6. What is your **marital status**? | | | Never married | | | | 1 | | | |  | | | |  |
|  |  |  | Currently married | | | | 2 | | | |  |  |  |  |  |
|  |  |  | Separated | | | | 3 | | | |  |  |  |  |  |
|  |  |  | Divorced | | | | 4 | | | |  |  |  |  |  |
|  |  |  | Widowed | | | | 5 | | | |  |  |  |  |  |
|  |  |  | Cohabitating | | | | 6 | | | |  |  |  |  |  |
|  |  |  | Refused | | | | 88 | | | |  |  |  |  |  |
| 7. Which of the following best describes your **main** **work** status over the past 12 months? | | | Government employee | | | | 1 | | | |  | | | |  |
|  |  |  | Non-government employee | | | | 2 | | | |  |  |  |  |  |
|  |  |  | Self-employed | | | | 3 | | | |  |  |  |  |  |
|  |  |  | Non-paid | | | | 4 | | | |  |  |  |  |  |
|  |  |  | Student | | | | 5 | | | |  |  |  |  |  |
|  |  |  | Homemaker | | | | 6 | | | |  |  |  |  |  |
|  |  |  | Retired | | | | 7 | | | |  |  |  |  |  |
|  |  |  | Unemployed (able to work) | | | | 8 | | | |  |  |  |  |  |
|  |  |  | Unemployed (unable to work) | | | | 9 | | | |  |  |  |  |  |
|  |  |  | Refused | | | | 88 | | | |  |  |  |  |  |
| 8. Which of the following best describes the place of your **main accommodation** over the past 12 months? | | | Guest house  Own house  Rental house  In street  Refused | | | | 1  2  3  4  88 | | | |  | | | |  |
| 9. How many people older than 18 years, including yourself, live in your household? | | | Number of people  Not applicable  Refused | | | | └─┴─┘  99  88 | | | |  | | | |  |
| 10. Taking the past year, can you tell me what the **average earnings** of the household have been? *(record only one, not all 3)* | | | Per week | | | └─┴─┴─┴─┴─┴─┴─┘ | | | | |  | | | |  |
|  |  |  | OR per month | | | └─┴─┴─┴─┴─┴─┴─┘ | | | | |  | | | |  |
|  |  |  | OR per year | | | └─┴─┴─┴─┴─┴─┴─┘ | | | | |  | | | |  |
|  |  |  | Refused | | | 88 | | | | |  | | | |  |
| **Risk Behaviors** | | | | | | | | | | | | |  | | |
| 11. How many **sexual partners** have you had in the past 6 months? ________ | | | | | | | | | |  | | |  | | |
| 12. How many times in the past six months have you **used a condom** when engaging in sexual practices? | | | Never | 1 | | --- | --- | | Sometimes | 2 | | Always | 3 | | Refused | 88 | | | | | | | | |  | | |  | | |
| 13. I would like to know if you have ever having injected non-medically authorized **psychotropic substances** in the past 30 days | | | Yes | 1 | | --- | --- | | No | 2 If No END | | Refused | 88 If Refused END | | | | | | | | |  | | |  | | |
| 14. How long have you injecting drugs | | | Less than one year | 1 | | --- | --- | | Around one year | 2 | | Around two years | 3 | | Around three years | 4 | | More than 3 years | 5 | | Refused | 88 | | | | | | | | |  | | |  | | |
| 15. In average **how often** do you use injected drugs per day in the last six months? | | | Once | 1 | | --- | --- | | Two times | 2 | | Three times | 3 | | More than three times | 4 | | Refused | 88 | | | | | | | | |  | | |  | | |
| 16. Have you ever enrolled in the **medication-assisted treatment** for harm reduction in the past 30 days? | | | Yes | 1 | | --- | --- | | No | 2 | | Refused | 88 | | | | | | | | |  | | |  | | |
| 17. Have you ever **exchanged drugs for sex** in the past 6 months? | | | Yes | 1 | | --- | --- | | No | 2 | | Don’t Know | 77 | | Refused | 88 | | | | | | | | |  | | |  | | |
| 18. Have you ever had **sex with someone you know injects drugs** in the past 6 months? | | | Yes | 1 | | --- | --- | | No | 2 | | Don’t Know | 77 | | Refused | 88 | | | | | | | | |  | | |  | | |
| 19. Have you ever shared the same needle with someone else to inject drugs in the last six months? | | | Yes | 1 | | --- | --- | | No | 2 | | Don’t Know | 77 | | Refused | 88 | | | | | | | | |  | | |  | | |
| **Status of HIV, HBV and HCV infection** | | | | | | | | | | | | |  | | |
| 20. Have you ever tested for HIV in the past six months? | | | Yes | 1 | | --- | --- | | No | 2 | | Don’t Know | 77 | | Refused | 88 | | | | | | | | |  | | |  | | |
| 21. Do you know your HIV status in the past three months? | | | Yes | 1 | | --- | --- | | No | 2 | | Don’t Know | 77 | | Refused | 88 | | | | | | | | |  | | |  | | |
| 22: If you know your HIV status, Please share with me. | | | Positive | 1 | | --- | --- | | Negative | 2 | | Refused | 88 | | | | | | | | |  | | |  | | |
| 23. Have you ever tested for Hepatitis B, or C or both in the past six months? | | | Yes | 1 | | --- | --- | | No | 2 | | Don’t Know | 77 | | Refused | 88 | | | | | | | | |  | | |  | | |
| 24. Do you know your status of Hepatatis B or C or both in the last six months | | | Yes for HBV | 1 | | --- | --- | | Yes for HCV | 2 | | Yes for Both | 3 | | Don’t know | 77 | | Refused | 88 | | | | | | | | |  | | |  | | |
| 25: If known, what is your status of HBV in the last six months. | | | Positive | 1 | | --- | --- | | Negative | 2 | | Refused | 88 | | Not applicable | 999 | | | | | | | | |  | | |  | | |
| 26: If known, what is your status of HCV in the last six months. | | | Positive | 1 | | --- | --- | | Negative | 2 | | Refused | 88 | | Not applicable | 999 | | | | | | | | |  | | |  | | |
